# Radiological Features and Management of Intracranial Aneurysms Associated With Moyamoya Disease: A Case Series of Single-Center Experience

**DOI:** 10.7759/cureus.52370

**Published:** 2024-01-16

**Authors:** Subash Phuyal, Shailesh B Gaikwad, Ajay Garg, Nishchint Jain, Manoj Nayak, Leve J Devarajan

**Affiliations:** 1 Neuroimaging and Interventional Neuroradiology, Upendra Devkota Memorial (UDM) National Institute of Neurological and Allied Sciences, Kathmandu, NPL; 2 Neuroimaging and Interventional Neuroradiology, All India Institute of Medical Sciences, New Delhi, New Delhi, IND; 3 Radiodiagnosis, All India Institute of Medical Sciences, Bhubaneswar, Bhubaneswar, IND

**Keywords:** aneurysmal subarachnoid hemorrhage, endovascular treatment, stroke, intracranial aneurysms, moyamoya disease (mmd)

## Abstract

Background

Moyamoya disease (MMD) can be a major cause of hemorrhagic stroke. Though extensive angiographic studies have been undertaken, the understanding of the association between aneurysms and MMD remains unanswered. In this study, we explore the association of the aneurysm with MMD and its management. We have also reviewed such associations described in the literature and how the present cases differ from those previously described.

Materials and methods

The clinical and radiologic data of moyamoya disease cases were accessed from medical and radiological records between January 2010 and July 2017. Two neuroradiologists independently analyzed the data and imaging details.

Results

Out of 103 patients with MMD, eight patients (7.77%) had associated intracranial aneurysms with eleven aneurysms. Out of the 11 aneurysms, five were the tip of the basilar artery aneurysms and were the most common location for aneurysm (45.5%), followed by lenticulostriate artery, PCA perforator, and distal ACA (DACA) in the P1 PCA, P2 PCA, and P3 PCA artery aneurysms. Out of eight patients, five (62.5%) had a hemorrhage on a non-contrast computed tomography (NCCT) scan of the brain, whereas three (37.5%) had an ischemic presentation. Out of 11 aneurysms, seven aneurysms, including three basilar tip aneurysms (unruptured) and one PCA perforator (ruptured), and three saccular PCA (P1, P2, and P3) (ruptured) were treated by endovascular coiling. Follow-up angiography showed stable aneurysmal occlusion except in one basilar tip, where recurrence was observed.

Conclusions

MMD-intracranial aneurysm is commonly observed in patients with intracranial hemorrhage and carries a higher risk of rupture. Therefore, identification of the aneurysm is essential for management. Endovascular treatment, either with coil or glue embolization, can be a safe and effective treatment method for such aneurysms with long-term good results.

## Introduction

Moyamoya disease (MMD) is one of the causes of hemorrhagic stroke. Although extensive angiographic studies have been undertaken, the understanding of the association between aneurysms and MMD remains unanswered. MMD is a rare cerebrovascular progressive steno-occlusive disease involving terminal internal carotid arteries (ICAs) and its branches with distinctive, abnormal collateral vascular channels, so-called moyamoya vessels, particularly present at the base of the brain [1-3]. Steno-occlusive change can affect both ICA and posterior cerebral artery (PCA) and is believed to be a neural crest cell derivative [3,4]. Clinically, MMD has both ischemic and hemorrhagic presentation, the latter being more common in older age groups. Intracranial aneurysms (IAs) can occur in patients with MMD. However, their significance for hemorrhagic presentation is not well documented. MMD-related IAs were first described in the 1970s [2,5], and, since then, these aneurysms have frequently been described in the literature. The pathogenic mechanism behind these MMD-aneurysms is complicated and multifactorial, which includes hemodynamic stress and pathological vascular architecture. Managing these MMD aneurysms is quite challenging because of their anatomical variations and pathological complexities. In a single-center retrospective study of MMD, we have attempted to identify the prevalence of aneurysms and their possible association with clinical presentation and patient outcomes.

## Materials and methods

A retrospective study was conducted between January 2010 and July 2017 in the neurosciences center of our institute. Patients with MMD were included in this study. All the patients were evaluated for associated IAs. MMD patients with vascular anomalies other than IAs or other vasculopathies were excluded from this study. The Institutional Ethics Committee (All India Institute of Medical Sciences, New Delhi, India) approved this study and waived the requirement to obtain informed consent from patients because of its retrospective design (IECPG-104/20.07.2017).

The neurovascular digital subtraction angiography (DSA) database in the Department of Neuroimaging and Interventional Neuroradiology was accessed for patients with MMD and associated IAs. Clinical and radiological files of these patients were obtained from the records section and the picture archiving and communication system (PACS). Data on demographics, clinical manifestations, neurological deficits, overall patient’s clinical illness at presentation and at the time of discharge from the hospital, and follow-up were collected from the records section (e.g., outpatient, in-patient records, and patient discharge summaries). Original hard copies of computed tomography (CT), magnetic resonance angiography (MRA), and DSA, as well as soft copies from PACS, were retrieved for evaluation. Two neuroradiologists independently analyzed the hard and soft copy images to access the IA.

The diagnosis of moyamoya was made based on characteristic radiographic findings involving the narrowing of the terminal segments of the ICA, often with the associated development of collateral vessels. The status of brain parenchyma (infract, hemorrhage, and atrophy) was reviewed.

At the time of diagnosis, a definitive treatment (either surgical or endovascular) was taken into consideration for ruptured aneurysms in compliance with the standard protocol followed at our institution. For unruptured aneurysms, routine follow-up was advised; however, further treatment would also be taken into consideration if a lesion grew larger or ruptured during follow-up.

For every patient included, there were available angiographic follow-up data (within the study duration). For both ruptured and unruptured aneurysms, routine angiographic follow-ups (MRA) were conducted at one, two, and four years. When further follow-ups were thought to be required, they were conducted according to each patient's unique clinical and radiological circumstances. These follow-up images were analyzed by two neuroradiologists for recurrence or rupture of aneurysms.

Data obtained were analyzed using Statistical Product and Service Solutions (SPSS) software (version 26.0; IBM SPSS Statistics for Windows, Armonk, NY). Only descriptive statistics are provided without any comparisons with other diseases or treatments because this study covered the demographics and radiological outcomes of patients with MMD-associated aneurysms. The information is displayed as mean ± standard deviation values for continuous variables and as percentage and number values for categorical variables.

## Results

**Figure 1:**
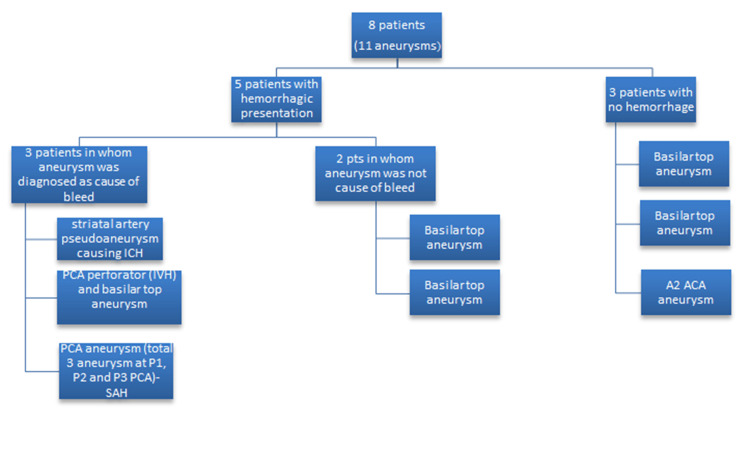
Distribution pattern of aneurysms in patients of moyamoya disease (MMD).

A neurovascular database search showed 103 patients with MMD. Out of 103 patients with MMD, eight (7.77%) patients had associated cerebral aneurysms. In these eight patients, a total of 11 aneurysms were identified. Of the 11 aneurysms, five aneurysms (45.4%) were the tip of basilar artery aneurysms. It was the most common location for aneurysm in our study. One aneurysm each in the lenticulostriate artery, posterior cerebral artery (PCA) perforator, distal anterior cerebral artery (DACA), and in the P1 PCA, P2 PCA, and P3 PCA arteries were seen in our study. These three PCA aneurysms were seen in the same patient (Figure 1).

The mean age was 42.8±10.83 years. Male and female were equal in number (M: F-1:1). Out of the eight patients, five patients had hemorrhage as initial presentation, whereas three patients had ischemia. Out of five patients with the hemorrhagic presentation, we could identify an aneurysm as the cause/site of bleed in only three patients. In these three cases, striatal artery pseudoaneurysm (hypertrophied basal collaterals) and PCA perforator aneurysm were identified as the cause of intraventricular hemorrhage (IVH) in two patients, whereas one patient with subarachnoid hemorrhage (SAH) had P1, distal P2, and P3 (total of three) PCA aneurysms (Table 1).

**Table 1:** Description of demographics, clinical presentation, and imaging findings in patients with MMD. MMD: moyamoya disease, IVH: intraventricular bleed, ICH: intracranial hematoma, SAH: subarachnoid hemorrhage, PCA: posterior cerebral artery, ACA: anterior cerebral artery, MCA: middle cerebral artery

Age (Y)	Sex	Clinical presentation	Imaging findings	Aneurysm type
45	F	Bleed (IVH)	Intraventricular bleed	Basilar top and lenticulostriate perforator aneurysm
34	F	Bleed (IVH)	Intraventricular bleed	Basilar top aneurysm
40	F	Bleed (ICH)	Intracerebral bleed	Basilar top aneurysm
43	M	Bleed (ICH)	Intracerebral bleed	PCA perforator aneurysm
45	M	Bleed (SAH)	Subarachnoid bleed	Left P1, distal P2 and P3 PCA aneurysm
55	M	Acute stroke	Acute watershed infarct in left MCA/PCA.	Basilar top aneurysm
41	M	Chronic stroke	Left frontal white matter infarct	Basilar top aneurysm
40	F	Chronic stroke	Chronic territorial infarct in right ACA	Left distal ACA aneurysm (DACA)

In the remaining two patients, basilar top aneurysms were seen and were not found to be the cause of hemorrhage as these patients were presented with IVH and intracranial hematoma (ICH), not with SAH.

In DSA, most patients (6/8) had complete occlusion of supraclinoid ICA. However, only a single patient had PCA (posterior circulation) involvement. All the patients had moderate to good basal and pial collaterals with poor dural collaterals. All patients had poor to moderate baseline perfusion with good augmentation by collaterals. The majority (5/8) of our cohorts had Suzuki grade III. The details of the DSA findings are described in (Table 2).

**Table 2:** Angiographic features of MMD complicated by aneurysms. B/L: Bilateral, ICA: internal carotid artery, MCA: middle cerebral artery, ACA: anterior cerebral artery, PCA: posterior cerebral artery

No	Side	ICA stenosis	MCA stenosis	ACA stenosis	PCA stenosis	Collaterals	Perfusion via ICA without collaterals	Perfusion via ICA plus collaterals	Suzuki grading
1	B/L	+	+	+	-	Basal > pial	<1/3rd	>2/3rd	IV
2	B/L	+	+	+	-	Basal > pial	<1/3rd	>2/3rd	III
3	U/L	+	-	+	-	Basal > pial	1/3^rd^-2/3rd	>2/3rd	III
4	B/L	+	+	+	+	Pial > basal	<1/3rd	>2/3rd	III
5	B/L	+	+	+	-	Basal, dural, pial	>2/3rd	>2/3rd	III
6	B/L	+	-	+	-	Basal = pial	1/3^rd^-2/3rd	>2/3rd	II
7	B/L	+	+	+	-	Basal, pial	<1/3rd	>2/3rd	III
8	U/L	+	+	+	-	Basal > pial	1/3^rd^-2/3rd	>2/3rd	II

**Figure 2:**
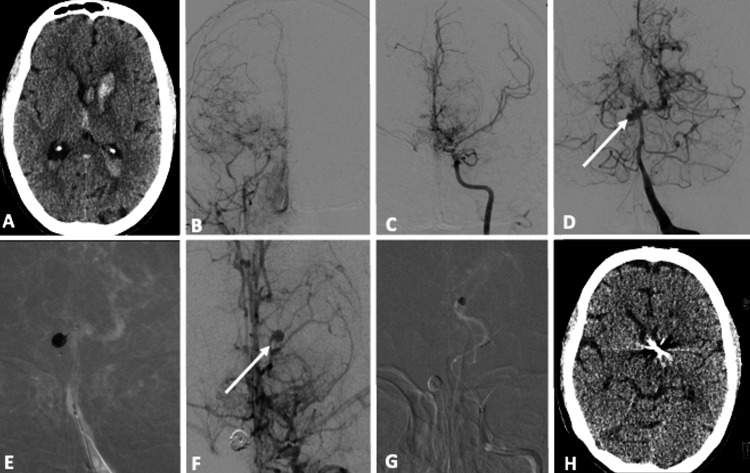
Noncontrast CT and DSA images pre- and post-coiling of a patient with multiple aneurysms with MMD (Case 1). Axial CT image (A) showing intraventricular bleed. Bilateral (B/L) internal carotid artery (ICA) angiogram (B&C) reveals attenuated B/L ICAs (R>L). The left vertebral artery (VA) angiogram (D) revealed a lobulated basilar top aneurysm, which was coil embolized using balloon assistance (E). The striatal pseudo-aneurysm was also catheterized through super-selective navigation through the posterior communicating artery perforator and was coiled (F and G). Post-embolization axial CT (K) was normal with no parenchymal infarct/bleed.

**Figure 3:**
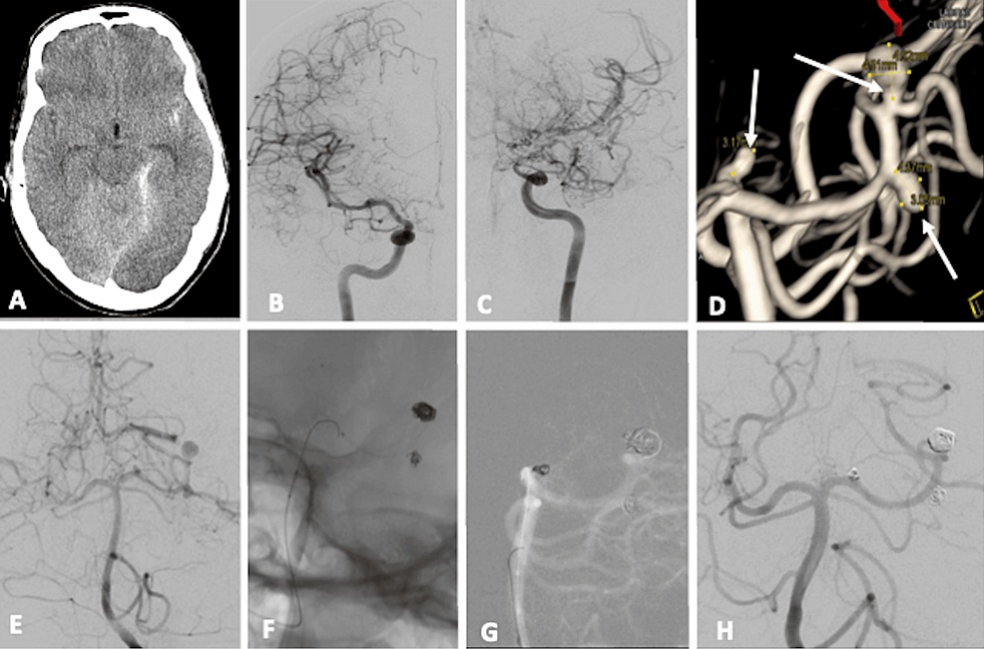
Noncontrast CT and DSA images pre- and post-coiling of a patient with multiple aneurysms with MMD (Case 5). Axial CT image (A) showed a subarachnoid bleed in the left Sylvian fissure and left tentorium cerebelli. B&C revealed a narrowing of bilateral supraclinoid ICA, A1 anterior cerebral artery (ACA), and left M1 middle cerebral artery (MCA). A 3D angiogram (D) and left vertebral artery (VA) angiogram (E) showed three flow-related aneurysms, one each in P1, distal P2, and P3 posterior cerebral arteries (PCA) (white arrows). All three aneurysms were coiled under balloon protection (F-H), with the exclusion of an aneurysm and good filling of left PCA on the control angiogram.

Out of the identified 11 aneurysms, seven aneurysms were treated: three basilar tip aneurysms (unruptured), one PCA perforator aneurysm (ruptured), and three saccular PCA (P1, P2, and P3) aneurysms (ruptured). All aneurysms were treated by endovascular coiling (Figures 2-3).

Follow-up angiography at one year showed stable aneurysmal occlusion except in one basilar tip aneurysm, wherein a recurrence of the aneurysm was noted.

## Discussion

In our study, 8/103 (7.77%) patients of MMD had 11 associated cerebral aneurysms. The basilar tip had commonly five (45.4%), followed by one aneurysm each in the lenticulostriate artery, posterior cerebral artery (PCA) perforator, distal anterior cerebral artery (DACA), and P1 PCA, P2 PCA, and P3 PCA arteries. Moreover, 5/8 (62.5%) patients had hemorrhage as the initial presentation, whereas 3/8 (37.5%) patients had ischemia. In DSA, most patients (6/8) had complete occlusion of supraclinoid ICA; however, only a single patient had PCA involvement. The majority 5/8 (62.5%) had Suzuki grade III. Additionally, 7/11 (63.6%) aneurysms were treated: three basilar tip aneurysms (unruptured), one PCA perforator aneurysm (ruptured), and three saccular PCA (P1, P2, and P3) aneurysms (ruptured) by the endovascular route. Follow-up angiography at one year showed stable aneurysmal occlusion, except in one basilar tip aneurysm wherein recurrence of the aneurysm was noted.

Suzuki and Takaku described this disease under “cerebrovascular ‘moyamoya’ disease,” in the Archives of Neurology in 1969 [1], and Takeuchi and Nishimoto termed it as “hypogenesis of bilateral internal carotid arteries” [3]. After this nomenclature, the word “moyamoya disease” was described throughout the world [6]. Recently, MMD is believed to be a vascular variant of neurocristopathy, which is derived from both cephalic and cardiac neural crest cells [7], and it was observed that ring finger protein (RNF) 213 is the key factor behind the regulation of angiogenesis [8]. Initially, MMD was noticed to be prevalent only in Japan [3], and now it is observed in many other countries [3,9]. MMD presenting with hemorrhage occurs more commonly in older age as compared to ischemic MMD and has a higher rate of rebleeding and mortality because of its aggressive course [10]. The pathogenesis behind the formation of MMD-related aneurysms is complicated and multifactorial, which includes hemodynamic disturbance, pathological angioarchitecture, and anatomical location of the aneurysm. The classification of MMD-related aneurysms varies because of complexities related to the mechanism of formation and its location. MMD-IAs are classified conventionally into various types depending upon their locations, such as involvement of major arteries or peripheral arteries [11,12]. However, Yeon et al. classified MMD-aneurysms into three subtypes that include aneurysms along major vessels like the circle of Willis (COW), along distal peripheral vessels like the anterior or posterior choroidal artery, and along with moyamoya collaterals [13]. Takahashi et al. divided different causes of hemorrhage in MMD into three reasons: hemodynamic stress (e.g., rupture of saccular aneurysms along major arteries involving COW), rupture of moyamoya vessels, and rupture of microaneurysms formed along moyamoya vessels [11]. Zhang et al. further classified the MMD-related aneurysms into major artery aneurysms (both anterior and PCA), peripheral artery aneurysms (both anterior and posterior choroidal artery), moyamoya vessel aneurysms (lenticulostriate and thalamoperforating artery), meningeal artery, and aneurysms at the site of anastomosis [14]. Because of these variations of MMD-IA, a relatively simpler classification was tried in our study based on pathomorphological features like hemodynamic true saccular aneurysms (HTA) and dissecting or pseudoaneurysms (DPA), which can present in collateral vessels in patients with MMD.

Hemorrhage in MMD occurs typically in the basal ganglia, thalamus, and commonly extends into the adjacent ventricles. Various theories behind MMD-related intracranial bleeding because of hemodynamic stress, which includes rupture of fragile moyamoya vessels, COW aneurysms, or deep collateral perforators pseudoaneurysms [15,16]. The change in hemodynamics because of gradual and progressive occlusion of the ICA in MMD results in a change in hemodynamic flow within other intracranial vessels causing reactive and compensatory dilation of the collateral vessels and, hence, increased blood flow through the vessels into the brain parenchyma. This hemodynamic disturbance frequently leads to aneurysm formation, and, subsequently, it can rupture, leading to intracranial hemorrhage [17]. The aneurysm formation in our case series of MMD in relatively older patients could be because of hemodynamic disturbance for a longer period. Aneurysm involving intracranial arteries or collateral vessels is infrequently identified on the cerebral angiography of a patient with MMD. It is of utmost importance for us to identify if the given aneurysm is the cause of hemorrhage or not as this has an important bearing on further clinical management.

The literature survey showed that the prevalence rate of IAs in MMD varies between 3% and 14% in adults [11,18-19]. The prevalence of aneurysms in MMD can be as high as 18% versus 2-3% in the general population [20]. In concordance with published literature, the absolute prevalence of aneurysms was low in our set-up MMD (7.8%). Most aneurysms were basilar tip aneurysms. In these cases, the possible mechanism for aneurysm formation could be because of the augmentation of anterior circulation flow in narrowed ICAs by hyperdynamic flow in the PCA. However, none of these aneurysms presented with acute/remote rupture. Perforator artery aneurysms are formed secondary to the rupture of basal collaterals leading to the formation of pseudoaneurysm. They represent the site for possible rerupture and should be excluded from circulation.

We found eleven aneurysms among MMD cases, who presented to our hospital (n=103), and five aneurysms of these eleven aneurysms presented with intracranial hemorrhage. The rate of aneurysmal hemorrhage in the cases of hemorrhagic MMD cases in our study cohort was 45.5%. Contrary to our study, a lower incidence of aneurysmal rupture (24.7%) was reported by Rhim et al. in 99 MMD patients who presented with hemorrhage [19]. From this data, MMD-related aneurysms can be inferred to play a vital role in MMD patients who can present with intracranial hemorrhage; however, larger studies would be required to conclusively state this.

Various treatment strategies like conservative, endovascular management, and surgical clipping for management in MMD-related IA have been described, depending on the anatomic location and underlying pathophysiology [21]. In our study, out of 11 aneurysms, seven aneurysms were treated: three basilar tip aneurysms (unruptured), one P1 PCA perforator aneurysm (ruptured), and three saccular PCA (P1, P2, and P3) aneurysms (ruptured). All aneurysms were treated by endovascular coiling, and follow-up angiography showed stable aneurysmal occlusion in all aneurysms except in one basilar tip aneurysm wherein recurrence of the aneurysm was observed. The possible explanation for the recurrence could be because of significant stenosis of bilateral ICAs, which requires more significant hyperdynamic flow from PCA.

A recent systematic review by Larson et al. in 275 patients with 311 aneurysms showed that 59.6% of aneurysms were located in COW, 33.7% in peripheral vessels, and 6.7% in other vessels [21]. In their study, IAs involving COW, 87.2% treated with endovascular procedures had a better outcome than 56.7% with open surgery, whereas 95% vs 69.6% for peripheral IA; however, 65.7% of peripheral IA had spontaneous resolution as compared with 12.0% COW IA with or without revascularization [21]. They conclude that MMD-IA is better managed with endovascular techniques both for COW and peripheral IA; however, surgical options can also be considered for anterior circulation of COW IA if endovascular treatment is not feasible.

Fragile collateral vessels with peripheral artery aneurysms are more prone to rupture; hence, early treatment is necessary [22]. However, this is difficult to manage because these vessels are fragile, deep in location, and small in caliber [23]. The later study showed the outcome was better with endovascular techniques than with open techniques. Larson et al. showed a resolution of aneurysms and reduced risk of bleeding after the revascularization procedure; however, the risk of rupture was there during the interval period [21]. Takahashi et al. showed PCA aneurysms needing early treatment than anterior circulation because of a relatively higher risk of rupture (17.1% per year vs 3.0% per year; hazard ratio, 5.83; 95% confidence interval, 1.60-21.27) [24].

Limitations

The study has certain limitations. First, the study included a relatively small sample size of eight patients associated with MMD-IAs. A larger sample size could provide more robust and generalizable results. Second, the study design is retrospective, relying on medical records and imaging data collected over seven years. Retrospective studies may be subject to selection bias and incomplete or inconsistent data. Third, the data for this study were obtained from a single medical center, which may limit the generalizability of the findings to a broader population with MMD. Fourth, the study reports a preference for endovascular coiling for the treatment of MMD aneurysms. This may introduce selection bias because treatment decisions were not randomized and could be influenced by the preferences and expertise of the treating physicians.

## Conclusions

MMD-associated intracranial aneurysms are commonly associated with hemorrhagic MMD and are prone to rupture because of hemodynamic disturbance. Therefore, the identification of an aneurysm is an important finding in patients with MMD. This has a bearing both on clinical presentation and management. Aneurysm formation in MMD is either because of hemodynamic factors or basal collateral rupture. Endovascular treatment, either with coil or glue embolization, can be a safe and effective treatment method for such aneurysms with long-term good results.
